# Evolution of chronic renal impairment and long-term mortality after de novo acute kidney injury in the critically ill; a Swedish multi-centre cohort study

**DOI:** 10.1186/s13054-015-0920-y

**Published:** 2015-05-06

**Authors:** Claire Rimes-Stigare, Paolo Frumento, Matteo Bottai, Johan Mårtensson, Claes-Roland Martling, Sten M Walther, Göran Karlström, Max Bell

**Affiliations:** Department of Anaesthesia, Surgical Services and Intensive Care (ANOPIVA) F2, Karolinska University Hospital, Solna, 171 76 Stockholm Sweden; Section of Anaesthesia and Intensive Care Medicine, Department of Physiology and Pharmacology, Karolinska Institute, Stockholm, Sweden; Unit of Biostatistics, Institute of Environmental Medicine (IMM), and Karolinska Institute, Stockholm, Sweden; Department of Intensive Care, Austin Hospital, Melbourne, VIC Australia; Department of Cardiothoracic Anaesthesia and Intensive Care, Linköping University Hospital, Linköping, Sweden; Division of Cardiovascular Medicine, Department of Medical and Health Sciences, Linköping University, Linköping, Sweden; The Swedish Intensive Care Registry, Karlstad, Sweden

## Abstract

**Introduction:**

Acute Kidney Injury (AKI) is common in critical ill populations and its association with high short-term mortality is well established. However, long-term risks of death and renal dysfunction are poorly understood and few studies exclude patients with pre-existing renal disease, meaning outcome for de novo AKI has been difficult to elicit. We aimed to compare the long-term risk of Chronic Kidney Disease (CKD), End Stage Renal Disease (ESRD) and mortality in critically ill patients with and without severe de novo AKI.

**Method:**

This cohort study was conducted between 2005 and 2011 in Swedish intensive care units (ICU). Data from 130134 adult patients listed on the Swedish intensive care register-database was linked with other national registries. Patients with pre-existing CKD (4192) and ESRD (1389) were excluded, as were cases (26771) with incomplete data. Patients were classified according to AKI exposure during ICU admission. Outcome in the de novo AKI group was compared to the non-exposed (no-AKI) intensive care control group. Primary outcome was all-cause mortality. Follow-up ranged from one to seven years (median 2.1 years). Secondary outcomes were incidence of CKD and ESRD and median follow-up was 1.3 years.

**Results:**

Of 97 782 patients, 5273 (5.4%) had de novo AKI. These patients had significantly higher crude mortality at one (48.4% vs. 24.6%) and five years (61.8% vs. 39.1%) compared to the control group. The first 30% of deaths in AKI patients occurred within 11 days of ICU admission whilst the 30-centile in the no-AKI group died by 748 days. CKD was significantly more common in AKI survivors at one year (6.0% vs. 0.44%) than in no-AKI group (adjusted incidence rate ratio (IRR) 7.6). AKI patients also had significantly higher rates of ESRD at one (2.0% vs. 0.08%) and at five years (3.9% vs. 0.3%) than those in the comparison group (adjusted IRR 22.5).

**Conclusion:**

This large cohort study demonstrated that de novo AKI is associated with increased short and long-term risk of death. AKI is independently associated with increased risk of CKD and ESRD as compared to an ICU control population. Severe de novo AKI survivors should be routinely followed-up and their renal function monitored.

**Electronic supplementary material:**

The online version of this article (doi:10.1186/s13054-015-0920-y) contains supplementary material, which is available to authorized users.

## Introduction

Acute kidney injury (AKI) is common amongst critically ill patients and incidence is rising in synchrony with an aging ICU population and their increasing comorbid burden [1-4]. The extremely high in-hospital mortality from AKI is well-established, but the extent to which survivors have a persistently increased risk of death, chronic kidney disease (CKD) and end-stage renal disease (ESRD) is uncertain [5-8].

Recent studies have demonstrated an association between AKI and the risk of developing CKD and ESRD. [9] Ponte and co-workers followed 187 AKI survivors and found that over 50% of patients, without pre-existing CKD, failed to recover their renal function [10]. A Danish study found AKI patients on renal replacement therapy (RRT) to have an 8.5% risk of developing ESRD, compared with 0.1% for ICU controls, corresponding to a hazard ratio (HR) of 105.6 [11]. Both CKD and ESRD are associated with elevated risk of death and reduced quality of life [12-17]. CKD is also specified by the World Health Organisation as a risk multiplier for cardiovascular disease, cancer, chronic respiratory disease, and diabetes [18,19]. Understanding renal recovery in AKI survivors is therefore essential in reducing long-term morbidity and mortality.

For a number of reasons the extent to which critically ill patients, with *de novo* AKI specifically, are at risk of CKD and ESRD is unknown. First, many studies lack pre-ICU data on CKD and hence, have not excluded patients with CKD. Therefore, it is difficult to differentiate the risks of developing CKD and ESRD in patients with true *de novo* AKI from those with acute on chronic kidney disease. Second, large studies have lacked ICU cohort comparisons, often studying AKI in heterogeneous populations such as hospital patients and therefore, generalisation of findings to ICU populations is problematic. Third, in those studies with long-term follow up of critically ill AKI survivors, mortality and ESRD but not CKD have been followed. No previous large-magnitude study has investigated CKD outcome in AKI survivors after intensive care treatment. Last, national data for outcome after AKI in Sweden have not previously been described.

The Swedish health care system maintains national databases of high quality and resolution, which offer the unique opportunity to reliably obtain data on comorbidity and outcome [20,21]. The Swedish Intensive Care Register (SIR) with almost complete national ICU coverage provided a population base and use of other national registers allowed us to identify and exclude patients with pre-existing CKD and ESRD. The present study aimed to determine short- and long-term outcomes, comparing risks of CKD, ESRD and mortality in patients with and without severe *de novo* AKI.

## Methods

### Study design

This cohort study used prospectively collected data from SIR and other Swedish national health registries. The Stockholm regional ethics committee (*Regionala etikprövningsnämnden i Stockholm*) approved the study and deemed informed consent unnecessary due to the large scale and observational nature of the study. Data were anonymized during crosslinking of registers.

### Study cohort

The SIR provided the population base for this study and data collected between January 2005 and January 2011 was extracted. We included the first admissions of all eligible adult subjects. Patients with CKD and ESRD prior to admission and those under 18 years, as well as those with incomplete records were excluded. Records were considered incomplete if International Classification of Diseases Version-10 codes (ICD-10), intervention codes and scoring systems were all absent because AKI classification of these patients was not possible.

SIR was established in 2001 and by 2005 was receiving data on ICU admissions from 41 of the then 88 Swedish ICUs. Recruitment has increased and SIR included 91% of all patients admitted to Swedish ICUs in 2011 [22]. Information is submitted from ICUs in district, county and tertiary referral centres and includes data from cardiothoracic, neurosurgical and burns injury units as well as general ICUs.

Mandatory SIR data includes patient characteristics and administrative details of each ICU admission. These data are complete. Submission of other variables is optional and these data are sometimes absent. Non-mandatory data include registration of interventions (for example, invasive ventilation or RRT), surgical status, complications and disease-severity scoring systems. Laboratory data were obtained from these scoring systems; premorbid laboratory data are not collated. Further details of SIR are found in Additional file 1.

SIR-data were cross-matched using the unique 10-digit Swedish identity number, assigned after birth or immigration with the following national registers [23]: 1) Swedish cause of death register, used to obtain details of all-cause mortality. This includes the deaths of all Swedish citizens and residents with a national identification number. This register is considered to be a very reliable source of data with over 99% of all deaths reported to it [24]; 2) National Patient Register (NPR) from which the cohort’s premorbid status was ascertained and classified according to the Charlson comorbidity index [25]. Patients with pre-existing renal disease (according to the Charlson definitions) were identified here, as were subjects developing CKD (ICD-10 codes N18 and N19) post ICU admission and 3) Swedish Renal Register (SRR) providing data on individuals with ESRD prior to and post ICU admission.

NPR is a mandatory register comprising the in- and outpatient registers; the ICD-10 diagnosis codes recorded here are used as the internal debit system within the Swedish health care system. The inpatient register contains all hospital discharges in Sweden, and complete coverage has been achieved since 1987 [26]. Outpatient register validation in 2011 indicated that 77% of all outpatient episodes were recorded, and private healthcare contacts accounted for the majority of omissions [27,28]. The SRR includes details of all individuals receiving treatment for ESRD. The database records the start and finish date for all patients receiving chronic haemodialysis or peritoneal dialysis and those who receive renal transplants. There are around 3,700 patients receiving dialysis in Sweden at any point in time. Our primary outcome measure was one-year mortality. Secondary endpoints were CKD and ESRD.

### Definitions

Premorbid creatinine levels or glomerular filtration rate (GFR) estimates were not available for this national cohort. Patients were considered to have *de novo* AKI (referred to as AKI from this point onwards) if at least one of the following criteria was fulfilled: 1) intermittent haemodialysis (IHD) or continuous renal replacement therapy (CRRT) was reported in SIR; 2) diagnosis of acute renal failure was recorded within the Acute Physiology and Chronic Health Evaluation (APACHE)-II score, defined as creatinine increased by more than 1.5 times from baseline, with urine output <410 ml in 24 hours; 3) the diagnosis code, acute kidney failure N17 in ICD10, was assigned at discharge, and 4) A serum creatinine >354 μmol/L (Kidney Disease Improving Global Outcomes (KDIGO) grade 3) was recorded on admission in APACHE-II, Simplified Acute Physiology Score (SAPS)-II or SAPS-III scoring systems. The ICU comparison cohort that did not fulfil these criteria, and did not have CKD or ESRD prior to ICU admission, was referred to as the no-AKI group.

CKD post ICU was considered present if ICD-10 codes N18 or N19 were assigned (NPR) at least 3 months after a patient’s ICU admission. ESRD post ICU was sought in SRR in survivors. Data from the Swedish cause of death register were available until 31 December 2011 and the maximum follow up for the primary outcome was 7 years. Data from other national registers were available until 31 December 2010 and therefore, secondary analysis was performed up to this date with a maximum follow up of 6 years.

### Statistical analysis

We report continuous data as median with the IQR. Categorical data are expressed as count and percentage. The Mann-Whitney test was used to compare distributions of continuous variables at baseline between AKI and non-AKI patients. Fisher’s exact test was used to compare prevalence of comorbidities between groups. A two-sided *P*-value <0.05 was considered significant.

### Primary analysis

We considered time from ICU admission to death or end of follow up (31 December 2011) for death or 31 December 2010 for secondary analysis or combined analysis), whichever occurred first. Information on emigration was unavailable. Survival curves were estimated by the Kaplan-Meier method and the log-rank test was used to verify equality of survivor functions between subgroups. We tested for proportionality of survival curves using Schoenfeld residuals and found evidence of non-proportionality; proportional hazard regression was therefore inappropriate and we instead used Poisson regression and present incidence rate ratios (IRRs).

### Multivariate analysis

Potential confounders were considered on the basis of prior knowledge of AKI and on whether addition of the covariates to the models changed estimates of relative risk by >10% [29]. We selected and tested age, sex, SAPS-III score (the scoring system most often recorded), acute surgery and the Charlson comorbidity groups as potential confounders and adjusted for these in our sensitivity analysis of subgroups. We present two models of multivariate analysis.

### Survival percentiles

Laplace regression was used to obtain the number of days of survival for the fifth to the thirtieth centiles of the AKI and non-AKI groups.

### Secondary analysis

This was performed in a similar manner to survival analysis. Time from admission to event (CKD or ESRD) was considered and censoring occurring at the point of ESRD in the case of CKD, or death or end of follow up whichever occurred first. A univariate model for the cumulative risk of developing either CKD, ESRD or of dying at any time point was constructed by considering the different endpoints of CKD, ESRD and death as competing events (Additional file 1: Figure S1). All analysis was performed using Stata version 12 (StataCorp LP, College Station, TX, USA).

## Results

**Figure 1:**
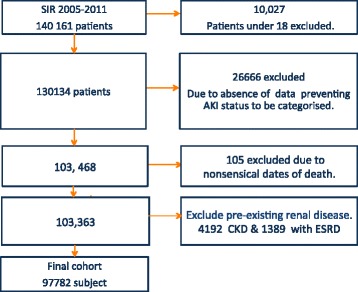
Flowchart of study population. SIR, Swedish intensive care register; AKI, acute kidney injury; CKD, chronic kidney disease; ESRD, end-stage renal disease.

The SIR included data on first admissions of 130,134 adult patients between January 2005 and January 2011. A flowchart detailing case exclusion is found in Figure 1. A total of 97,782 patients were included in the final analyses. Baseline characteristics and outcome for patients excluded due to insufficient data are presented in Additional file 1: Table S1. Median follow up for primary outcome was 2.1 years. Whilst for secondary outcome it was 1.3 years.

Of 97,782 patients 5,273 (5.4%) were identified as having AKI. The characteristics of patients with and without AKI are presented in Table 1. AKI patients were older (median 67.9 versus 59.3 years, *P* <0.001), had longer ICU length of stay (LOS) and were more often men (60.4% versus 56.4%, *P* <0.001). The AKI group had higher potassium (median 4.7 versus 4.1 mmol/l, *P* <0.001) and lower sodium (median 133 compared to 136 mmol/l, *P* <0.001) than controls. AKI patients were more acidotic (median pH 7.29 versus 7.36, *P* <0.001) with lower serum bicarbonate values at admission. APACHE II, SAPS-II and SAPS-III scores were all significantly higher in AKI patients.

**Table 1:** **Baseline characteristics of the study patients**

**Baseline characteristics**	**No acute kidney injury (n = 92,509)**	**Acute kidney injury (n = 5,273)**	***P*** **-value**
Mean age, years	59.3 (19.6)	67.9 (14.2)	<0.001
Median age, years	63 (45 to 75)	70 (61 to 78)	
Length of ICU stay, hours	23 (12 to 53)	68 (26 to 189)	<0.001
Women	40411 (43.6)	2086 (39.6)	<0.001
Number of admissions per patient, mean (SD)	1.31 (0.89)	1.29 (0.74)	0.19
Number of admissions per patient, median (IQR)	1 (1 to 1)	1 (1 to 1)	
Highest potassium, mmol/L	4.1 (3.9 to 4.5)	4.7 (4.1 to 5.5)	<0.001
Number	16069	1605	
Highest sodium, mmol/L	139 (136 to 142)	137 (134 to 141)	<0.001
Number	16121	279	
Lowest sodium, mmol/L	136 (133 to 139)	133 (130 to 137)	<0.001
Number	16121	1604	
Highest bilirubin, μmol/L	10 (6 to 17)	14 (8 to 26)	<0.001
Number	16863	1768	
Lowest arterial pH	7.36 (7.30 to 7.41)	7.29 (7.19 to 7.38)	<0.001
Number	40449	3968	
Lowest bicarbonate, mmol/L	23 (20 to 25)	18 (14 to 22)	<0.001
Number	3662	593	
Maximum creatinine (μmol/L)	80 (63 to 105)	254 (164 to 422)	<0.001
Number	47441	4076	
Maximum urea (mmol/L)	6.7 (4.3 to 10.1)	17.5 (11 to 26)	<0.001
Number	2717	532	
Lowest thrombocyte count, x 10^9^/L	230 (170 to 296)	199 (123 to 291)	<0.001
Number	32586	2695	
Apache II score	14 (8 to 20)	25 (20 to 32)	<0.001
Number	17231	1620	
SAPS II score	25 (0 to 41)	55 (42 to 70)	<0.001
Number	15376	764	
SAPS III score	52 (43 to 63)	68 (59 to 77)	<0.001
Number	18326	1437	
Intervention, number (%)^a^			
Invasive mechanical ventilation, number^a^	7155 (7.7)	994 (18.9)	<0.001
Acute surgery^a^	7282 (7.8)	662 (12.6)	<0.001
Elective surgery^a^	5237 (5.7)	295 (5.6)	1.0
Charlson comorbidity score, mean^b^ (SD)	1.78 (2.2)	2.64 (2.5)	<0.001
Comorbidity, number (%)			
Myocardial infarction	11896 (12.9)	986 (18)	<0.001
Congestive cardiac failure	12521 (13.5)	1324 (25.1)	<0.001
Peripheral vascular disease	8921 (9.6)	704 (13.4)	<0.001
Cerebrovascular disease	15658 (16.9)	808 (15.3)	<0.001
Dementia	1840(2.0)	99(1.9)	0.612
Chronic obstructive pulmonary disease	12999 (14.1)	808 (15.3)	0.010
Rheumatological disease	3311 (3.6)	269 (5.1)	<0.001
Peptic ulcer disease	5693 (6.2)	423(8.0)	<0.001
Cancer	15726 (17.0)	1262 (24.0)	<0.001
Metastatic disease	1918 (3.6)	304 (4.7)	<0.001
Mild liver disease	45021 (4.9)	369(7.0)	<0.001
Moderate or severe liver disease	3319 (2.1)	249 (5.8)	<0.001
Uncomplicated diabetes	13168 (14.2)	1372 (26.2)	<0.001
Diabetes with complications	4660 (5.0)	523 (9.9)	<0.001
Paraplegia	1787 (1.9)	90 (1.7)	0.287
HIV	125 (0.14)	4 (0.08)	0.328

Values are median (IQR) or n (%). if not otherwise stated. Information on laboratory data were obtained from the severity scorings systems APACHE II, SAPS II and SAPS III. APACHE and SAPS2 record the highest or lowest values recorded during the first 24 hours of ICU admission, and SAPS3 records values from 1 hour before until 1 hour after ICU admission. Values for scoring systems were not available in all patients; number denotes the number of patients in which this information was recorded. ^a^Intervention codes were also under-reported and therefore the number of patients in whom these data were available is detailed in the table. Reporting of all other baseline characteristics is complete. ^b^Charlson score is not age-adjusted. APACHE, acute physiology and chronic health evaluation; SAPS, simplified applied physiology score.

Interventions were under-reported, but AKI patients differed from controls by having higher rates of invasive ventilation (18.9 versus 7.7%, P <0.001) and emergency surgery (12.6 versus 7.8%, *P* <0.001).

Compared with the no-AKI group, patients with AKI had significantly more comorbidities except for dementia, and higher Charlson scores (1.78 versus 2.65, *P* <0.001). The proportion of patients with cardiovascular disease was higher in the AKI group, with 18.7% incidence of myocardial infarction and 25% incidence of congestive cardiac disease, compared to 12.9% and 13.5%, respectively, in the non-AKI patients. Diabetes was significantly more common in those with AKI (uncomplicated 26.3% versus 14.2%), likewise the proportion of AKI patients with liver disease and cancer was significantly higher than in the corresponding no-AKI group.

### Primary outcome

During follow up 34,473 patients (35.3%) died. Rates of all-cause mortality were higher in patients with AKI, 0.387 deaths per person/year compared to 0.134 in the non-AKI population with a mortality rate ratio (MRR) of 2.87. This diminished but remained elevated in both adjusted models presented (2.14 in model 1 and 1.15 in model 2, Table 2).

**Table 2:** **Multivariate regression analysis **

**A. Multivariate poisson regression analysis showing the association between acute kidney injury and mortality**							
**Group**	**Number**	**Deaths**	**Person-years**	**Mortality rate deaths/person-year (95% CI)**	**Crude MRR (95% CI)**	**Adjusted MRR** ^**a**^ **(95% CI)**	**Adjusted MRR** ^**b**^ **(95% CI)**
No AKI	92,509	31,530	2.3 x 10^5^	0.135 (0.134, 0.137)	1	1	1
AKI	5,273	2,943	7.6 x 10^3^	0.387 (0.374, 0.402)	2.87 (2.76, 2.97)	2.14 (2.06, 2.22)	1.15 (1.09, 1.21)

^a^Model 1: adjusted for age, gender, myocardial infarction and diabetes mellitus with complications. ^b^Model 2: adjusted for age, gender, simplified acute physiology score-III, myocardial infarction, cerebrovascular disease, diabetes mellitus with complications, moderate to severe liver disease, cancer and dementia. AKI, acute kidney Injury; MRR, mortality rate ratio.

Mortality rates were highest during the first days after admission. The AKI group had a significantly higher mortality rate than the comparison no-AKI group as illustrated in Table 2. Crude survival for the 5th to 30th centiles is presented in Table 3. Notably 5% of all patients died within one day of ICU admission. Twenty percent of AKI-patients were dead within 4 days of admission and the first 20% of deaths in the non-renal disease group occurred by 118 days. In the no-AKI group 30% had died by day 748 (2 years). In contrast 30% of patients in the AKI group had died within 11 days. A multivariate model (Additional file 1: Table S4) demonstrates how survival centiles differ depending on covariate patterns.

**Table 3:** **Crude survival centiles according to AKI status**

**Group**	**Crude Survival (days) for each given centile (95% CI)**			
	**5th**	**10th**	**20th**	**30th**
No AKI	1.0	5.9	118.5	748
AKI	0.63	1	4	11

AKI, acute kidney Injury; MRR, mortality rate ratio.

**Figure 2:**
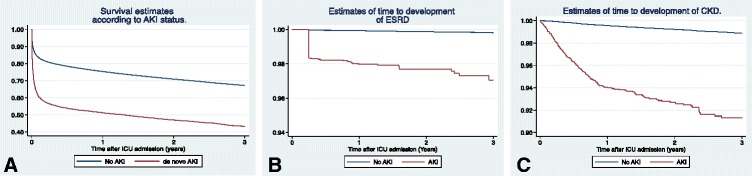
Estimates of survival, end-stage renal disease (ESRD) and chronic kidney disease (CKD) three years after ICU admission in patients with acute kidney injury (AKI) and patients with no AKI. Kaplan-Meier curves for cumulative mortality **(A)**, ESRD **(B)**, and CKD **(C)**. The steep drop observed at 90 days **(B)** reflects that most diagnoses of ESRD were made according to diagnostic guidelines after three months of persistent severely reduced glomerular filtration and dialysis dependence.

Kaplan Meier estimates showed 90-day survival in the AKI group to be 56.5% versus 80.6% in no-AKI comparison cohort (Figure 2A). One-year survival was 51.3% in those with severe AKI and 75.4% in the comparison no-AKI group. Five-year survival decreased to 38.2% for AKI-patients and 60.8% for the no-AKI cohort (Additional file 1: Table S2).

### Secondary outcomes

Incidence of CKD was significantly higher in the AKI group (6.0% at one year and 10.5% at 5 years) compared to controls (0.44% at 1 year and 1.8% at 5 years) with a crude IRR of 10.3 and an adjusted IRR of 7.6 (Table 4).

**Table 4:** **Multivariate poisson regression for secondary outcomes**

	**Multivariate poisson regression analysis**					
**Group**	**Patients, number**	**Events, number**	**Person-years**	**IR event/person-year (95% CI)**	**Crude IRR (95% CI)**	**Adjusted IRR** ^**a**^ **(95% CI)**
**A. CKD**						
No AKI	92,509	649	1.7 × 10^5^	0.0038 (0.0035, 0.0041)	1	1
AKI	5,273	193	4.9 × 10^3^	0.0340 (0.0341, 0.0452)	10.3 (8.8, 12.1)	7.6 (5.5, 10.4)
**B. ESRD**						
No AKI	92,509	116	1.7 × 10^5^	0.0007 (0.0006, 0.0008)	1	1
AKI	5,273	65	5.2 × 10^3^	0.0125 (0.0098, 0.0160)	18.6 (13.7, 25.2)	22.5 (12.9, 39.1)

^a^Adjusted for simplified applied physiology score version III, age, gender and diabetes. CKD, chronic kidney disease; ESRD, end-stage renal disease; AKI, acute kidney injury; IR, incidence rate; IRR, incidence rate ratio.

The risk of ESRD after ICU admission was low but significantly higher in the AKI group. The incidence rate in AKI patients was 0.0125 events per person-year compared to 0.0007 in the no-AKI cohort. The crude IRR was 18.6 (13.7, 25.2) and 22.5 (12.9, 39.1) after adjustment. The incidence of ESRD in the AKI group was 2.0% at 1 year increasing to 3.9% at 5 years, whereas in the the no-AKI group the 1-year and 5-year incidence of ESRD was 0.08 and 0.3%, respectively (Additional file 1: Table S3).

## Discussion

In the largest cohort of critically ill patients to examine the risk of CKD, ESRD and long-term mortality after *de novo* AKI, we showed that *de novo* AKI is independently associated with increased mortality and risks of ESRD and CKD. We demonstrated that patients with *de novo* AKI had 2.7 higher risk of dying, seven-fold risk of developing CKD and twenty-two times higher risk of developing ESRD than the comparison non-AKI cohort.

*De novo* AKI patients were identified using criteria aimed to detect patients with severe *de novo* AKI (described in Methods). These criteria included creatinine levels in line with classification of risk, injury, failure, loss, end-stage renal failure (RIFLE)-3, or AKI requiring dialysis, but also included the diagnoses of acute renal failure in APACHE II and acute kidney injury in ICD10, which could potentially have identified patients with somewhat milder AKI. However, diagnoses were generally under-reported and we suggest that cases of severe disease were more likely to have been recorded. We therefore believe that our cohort largely consists of cases of severe *de novo* AKI.

Our findings are largely consistent with other Scandinavian studies, investigating outcome after severe AKI. The Swedish Intensive Care Nephrology Group (SWING) study, conducted in 32 Swedish ICUs between 1995 and 2004, recorded 90-day mortality of 50.6% amongst patients who received CRRT [30]. This is somewhat higher than in our current study (43.5%). The FINNAKI study recorded 90-day mortality of 39% in patients classified as Acute Kidney Injury Network (AKIN) and KDIGO stage 3 [31]. The differing mortality may partly be explained by our AKI cohort being somewhat older (median age 68.3 versus 66 years) with higher SAPS-II scores (53.7 versus 43) during the same study period of 2006 to 2008 (not shown). Gammelager and colleagues in a Danish ICU-cohort recorded 90-day mortality in patients with RIFLE-F of 54.7%, and one-year mortality in the Laeknabladids Icelandic study was 48% [11,32].

International studies reporting long-term mortality for severe AKI vary from 48 to 76% at 1 year, to 84% at 5 years [7,33-35]. Recently, investigators in the RENAL-trial reported a total mortality rate of 63% in extended follow -up (up to 4 years) of 90-day survivors of AKI requiring dialysis [36]. This is slightly higher than in our Swedish cohort (58.1%).

In our current study the incidence of ESRD was lower than in the earlier Swedish SWING study (8.3% in those who had received CRRT) despite all-cause mortality being lower. However, patients with CKD were excluded from the present study. This result may indicate that treatment has improved since the SWING-study a decade ago [30]. The incidence of ESRD in SIR is lower than in the Gammelagers Danish cohort (8.5% at 6 months) and interestingly lower than in FINNAKI where 11.5% of survivors were dialysis-dependent [11,37]. This could reflect competing risks of death and ESRD. The Post RENAL study with a mean follow up of 42 months reported ESRD incidence of between 5.1 and 5.8% (depending on CRRT intensity) [36]. The Pannu Canadian cohort study of patients with KDIGO grade 2 or more recorded a very low incidence (2.1%) of dialysis dependence in survivors up to 34 months; these findings are comparable to our own [38].

CKD was significantly more common amongst AKI survivors than in the non-AKI group. These findings are consistent with other studies [9,10,39]. Renal dysfunction is common after AKI and CKD has been shown to occur in between 20 and 40% of patients [1,40,41]. As routine surveillance of renal function in AKI survivors is lacking in Sweden, the true extent of renal dysfunction in this group has been unknown [42]. A recent study found that follow up by nephrologists improved all-cause mortality in survivors with severe AKI [43]. Given the known association of renal dysfunction with reduced quality of life and increased risk of death, our findings underline the importance of post-ICU renal function surveillance and optimisation.

This study has limitations. First, under-reporting of diagnosis codes and particularly interventions in SIR hindered our ability to identify all severe AKI patients and to describe AKI incidence in this population. Under-reporting is a problem inherent in register research and particularly in cohorts of this magnitude. We suggest that although our method may not have exhaustively identified all patients with AKI, those assigned to the AKI group were correctly classified. The statistically significant difference in outcomes observed between groups and the similarity of our findings to other studies suggest that the identification process was largely successful.

Individuals excluded due to lack of sufficient data were younger, with lower comorbidity and disease severity scores and had shorter LOS than the study cohort. ICU admission generally occurred early during the study and was more likely to have been to a smaller ICU. Excluded subjects may represent a healthier group, with lower incidence of AKI. Higher turnover of healthier patients and initiation issues may explain failure to fully register data. Sicker individuals with longer LOS, like AKI-patients, may have been more conscientiously reported. Excluded patients had significantly lower mortality rates than the population studied. Their exclusion should have the effect of reducing differences between groups in the final cohort towards the null. Still, significant differences between AKI and non-AKI groups were observed.

The reliability of the NPR allowed us to identify patients previously diagnosed with CKD, however, baseline creatinine or GFR measurements were not available for the cohort and it is possible that some patients had undiagnosed CKD prior to admission to ICU.

Scoring systems are routinely presented in regression models and we include SAPS-III in one of our models for completeness. However creatinine is one of the variables used to derive the SAPS-score, which itself is used to classify patients into AKI and non-AKI groups. We suggest that use of SAPS-III as a covariate without removal of renal points could lead to over-adjustment. Furthermore, covariates such as age, acute surgery and malignancy are also common to SAPS-III and may compound issues of over-adjustment.

Although the maximum follow up was 7 years and 6 years for primary and secondary outcomes, respectively, complete follow up was only available for 1 year. It should therefore be noted that figures for 5-year survival and prevalence of ESRD and CKD are estimates.

Finally, data on emigration were unavailable, therefore, censoring due to emigration is not included in our regression analysis. Given the health status and median age of our cohort, we suspect that very few people are likely to have emigrated during the follow-up period and results are unlikely to be significantly affected.

The major strengths of our study are its magnitude, combined with the high coverage of the national intensive care cohort in the SIR database. Furthermore, the reliability of data on death, CKD and ESRD allows us to describe the long-term outcome in this large cohort with a uniquely high degree of internal validity. The cohort size permitted us to establish an ICU control population for comparison, which only a handful of previous studies have been able to do. This may make the external validity of our results higher than that of most of the previous studies.

### Significance

Our results are unique because in a large cohort, they demonstrate that mortality and rates of ESRD are relatively low compared to other contemporary studies and with an earlier Swedish study. This could reflect improvements in treatment of AKI. Our finding of an association between AKI and the risk of developing CKD has implications for clinical practice and ought to prompt routine nephrology follow up of these individuals. Using this database we intend to further investigate how outcomes for patients with pre-existing CKD and ESRD differ from those with *de novo* AKI and no AKI.

## Conclusion

This large Swedish cohort study has demonstrated that ICU patients with *de novo* AKI have elevated risk of death, chronic and end-stage renal disease as compared to an ICU control population. These findings emphasize the importance of monitoring and optimisation of renal function in survivors recovering from AKI. The total risks of death and ESRD are lower during this period as compared to a Swedish study conducted during the preceding decade and may indicate a temporal improvement in AKI management.

## Key messages

- AKI in critically ill patients is associated with an increased long-term risk of death.
- AKI survivors have a seven-fold increased risk of developing chronic kidney disease compared to non-AKI ICU patients.
- AKI survivors have twenty-two times increased risk of developing end-stage kidney disease compared to non-AKI ICU patients.
- Risk of death and ESRD after AKI may have improved over the last decade.

## Additional file

Additional file 1:
**Part A.** Details of the Swedish Intensive Care Register. Part B. Summary of details of excluded cases. **Table S1.** Comparison of characteristics and outcome of excluded patients. Part C. **Table S2.** Time-specific survival probability according to acute kidney injury (AKI) status. **Table S3.** Time-specific probability of developing end-stage renal disease (ESRD) and chronic kidney disease (CKD) in the AKI and no-AKI groups. **Table S4.** Adjusted survival centiles. **Figure S1.** Estimates of cumulative risk of developing CKD, ESRD or of death in patients with AKI.

## Abbreviations

AKI: acute kidney injury
APACHE: acute physiology and chronic health evaluation
CKD: chronic kidney disease
CRRT: continuous renal replacement therapy
ESRD: end-stage renal disease
GFR: glomerular filtration rate
HR: hazard ratio
ICD10: International classification of disease version 10
IHD: intermittent haemodialysis
IRR: incidence rate ratio
KDIGO: Kidney Disease Improving Global Outcomes
LOS: length of stay
MRR: mortality rate ratio
NPR: National Patient Register
RRT: renal replacement therapy
SAPS: simplified acute physiology score
SIR: Swedish Intensive care register
SRR: Swedish Renal register
SWING: Swedish Intensive Care Nephrology Group
