# Supplementary material for: Evolution of chronic renal impairment and long-term mortality after de novo acute kidney injury in the critically ill; a Swedish multi-centre cohort study
Source: Crit Care. 2015 May 6;19(1):221. doi: 10.1186/s13054-015-0920-y (PMC4457200; doi:10.1186/s13054-015-0920-y)
Supplement: Additional file 1: — Part A. Details of the Swedish Intensive Care Register. Part B. Summary of details of excluded cases. Table S1. Comparison of characteristics and outcome of excluded patients. Part C. Table S2. Time-specific survival probability according to acute kidney injury (AKI) status. Table S3. Time-specific probability of developing end-stage renal disease (ESRD) and chronic kidney disease (CKD) in the AKI and no-AKI groups. Table S4. Adjusted survival centiles. Figure S1. Estimates of cumulative risk of developing CKD, ESRD or of death in patients with AKI. [file 13054_2015_920_MOESM1_ESM.pdf]

Additional file 1.

Content:

Part A.

- Details of the Swedish intensive care register. Page 1

Part B.

- Summary of details of excluded cases. Page 2
- Table 1. Comparison of characteristics and outcome of excluded patients. Page 3 and 4.

Part C

- Table 2. Time specific survival probability according to AKI status. Page 5
- Table 3. Time specific probability of developing ESRD and CKD in AKI and No AKI groups. Page 5
- Table 4. Adjusted survival centiles. Page 6
- Figure 1. Estimates of cumulative risk of developing CKD, ESRD or of death in patients with AKI. Page 6.

A

Details of the Swedish intensive care register.
The Swedish intensive care register (SIR) uses national personal identification numbers to identify patients in the register and admissions to ICU without valid national personal identification are assigned a temporary number. SIR data is collected prospectively and transferred electronically to the register. A validation script identifies logical defects and returns these to each centre for re-evaluation prior to data being entered in the master database.

During the course of the study SIR used three disease severity scoring systems., including APACHE II (Acute physiology and Chronic health evaluation) used between 2008-2010, SAPS (Simplified Acute Physiology Score) version II (2005 - 2009) and SAPS III (2008-2011). Additionally the admission reason according to APACHE recorded on ICU entry and ICD-10 diagnoses determined at discharge, are available in many but not all patients.

B.

Summary of details of excluded cases.

Analysis showed that these ineligible patients were somewhat younger and had significantly shorter lengths of ICU stay. The incidence of all Charlson comorbidity groups was significantly lower than in the remaining cohort with the exception of HIV infection The percentage of deleted records was highest in first years of the study and in 2006, 59% of data was excluded. As reporting improved the percentage of patients with incomplete records decreased rapidly and represented 0·6% of the 2010 cohort. Quality of reporting varied initially between intensive care units with 6 units failing to report sufficient information in more than 20% of their cases. One year mortality for the excluded patients was significantly lower than for the remaining cohort (23·7% versus 27·1% respectively ($p<0.000$) and rates of ESRD did not differ from the overall ICU population.

Table 1. Comparison of characteristics and outcome of patients excluded due to insufficient data versus the included cohort.

Baseline characteristics	Patients included in the study. N= 103,363	Excluded Cases. N = 26666	P
Age (years) mean	60·3	59·0	<0.001
Age (years) median	64	62	
Length of ICU stay (hours) median	24	20	<0.001
Female (%) N= 44,480	43·0%	43·9	0·008
Number of admissions (mean)	1·31	1·33	<0.001
Comorbidity (%)			
Myocardial infarction N=14605	14·1	12·5	<0.001
Congestive cardiac failure N= 16281	15·8	14·06	<0.001
Peripheral vascular disease N= 10948	10·6	9·9	<0.001
Cerebro-vascular disease N=17742	17·2	16·06	<0.001
Dementia N= 2070	2·0	1·94	0·52
COPD N=14841	14·4	11·95	<0.001
Rheumatological disease N=4006	3·9	3·41	<0.001
Peptic ulcer disease N= 6729	6·5	6·08	<0.001
Cancer N= 18175	17·6	15·1	<0.001
Metastatic disease N= 3747	3·6	2·85	<0.001
Mild Liver disease N=5272	5·1	4·7	<0.001
Moderate or severe lever disease N= 2436	2·4	2·06	<0.001
Uncomplicated Diabetes N=16664	16·1	14·32	<0.001
Diabetes with complications N=6756	6·5	5·76	<0.001
Paraplegia N=2004	1·9	1·53	<0.001
HIV N=137	0·13	0·15	<0.001
Renal disease	5·4	4·3	<0.001
Outcome (%)			
Died 1 year (all cohort 27.06)	27·06	23·7	<0.001
ESRD	0·51	0·56	0·34
1-year mortality by admission year (%)			
2005	25·9	24·9	0·2
2006	31·5	23·6	<0.001
2007	28·9	23·7	<0.001
2008	26·8	18·8	<0.001

**Table 1 continued**<br>**Baseline characteristics**	**Patients included in the study.**<br>**N= 103,363**	**Excluded Cases.**<br>**N = 26666**	**P**
2009	26·9	23·2	0·48
2010	26·0	15·4	0·02
No of cases by admission year, N (%)			
2005	177007 (86%)	2775 (24%)	
2006	8328 (40·8%)	12064 (59·2%)	
2007	9598 (46%)	11291 (54%)	
2008	20248 (98·9%)	234 (0·1%)	
2009	23100 (99·6)	95 (0·4%)	
2010	25011 (99.4)	156 (0.6%)	

COPD= Chronic obstructive pulmonary disease, AKI = Acute Kidney Injury.

C

Table 2. Time specific survival probability according to AKI status·

Group	Survival probability (%)											
	30 day	CI	90 day	CI	180 day	CI	1 year	CI	3 year	CI	5 year	CI
No AKI	84·0	83·8-84·3	80·6	80·4-80·9	78·5	78·4-78·7	75·4	75·1-75·6	67·2	66·9-67·5	60·8	60·5-61·3
AKI	61·9	60·6-63·2	56·5	55·1-57·8	54·0	52·6-55·3	51·3	50·0-52·6	43·2	41·8-44·7	38·2	36·4-40·0

CKD= Chronic Kidney disease· ESRD= End Stage Renal disease· AKI= Acute kidney Injury· CI= 95% Confidence interval.

Table 3. Time specific probability of developing ESRD and CKD in AKI and No AKI groups.

Group	Outcome	Estimate of percentage of patients who develop outcome at specific time points·							
		6 months	CI	1 year	CI	3year	CI	5 year	CI
No AKI	ESRD	0·05	0·04-0·07	0·08	0·06-0·10	0·20	0·16-0·23	0·3	0·25-0·38
AKI		1.8	1.4-2.4	2.0	1.6-2.7	3.0	2.2-4.0	3.9	2·7-5.5
No AKI	CKD	0·21	0·18-0·24	0·44	0·39-0·49	1.1	1.0-1.2	1·8	1·6-1·9
AKI		3·7	3·0-4·4	6.0	5.1-7.0	8.7	7.5-10.2	10.5	13.0

CKD= Chronic Kidney disease· ESRD= End Stage Renal disease· AKI= Acute kidney Injury· CI= 95% Confidence interval.

Table 4. Adjusted survival centiles. Values show how estimated survival in days differs for the first 30 percent of patients according to covariate patterns.

Group	Adjusted model * Survival percentiles difference versus reference values					
	10th	CI	20th	CI	30th	CI
Male (Referent)<br>Female	0<br>13.6	 2.4 -25.0	0<br>10.5	 -4.8- 25	0<br>9.6	 -7.5-26.6
No AKI (Referent)<br>AKI	0<br>-22.9	 -41.7- -4.3	0<br>-36.2	 -58.5-13.8	0<br>-42.2	 -69.5- -14.9
Age: 60 years (Referent)<br>Every 1 year increase in age	0<br>-8.6	 -9.1- -8.1	0<br>-13.6	 -14.1-13.0	0<br>-16.3	 -16.9- 15.6
SAPS III score: 54 points (Referent)<br>Every one unit increase in score	0<br>-12.6	 -13.2- -11.9	0<br>-19.3	 -20- -18.6	0<br>-23.2	 -23.9- -22.5
Diabetes uncomplicated	-21.3	-36.6- -6.1	-26.3	-48.5- -4.0	-30.4	-54.3- -6.5
Diabetes with complications	-16.5	-40.0-7.0	-16.5	-46.6-13.5	-40.7	-72.2- -9.3
Myocardial infarction	-21.0	-33.6- -8.0	-35.8	-54.0- -18.6	-60.5	-79.9--41.1

AKI= Acute kidney injury. CI= 95% Confidence interval. SAPS = Simplified Physiology Score. *Model adjusted for sex, age, SAPS3 score, myocardial infarction and Diabetes Mellitus.

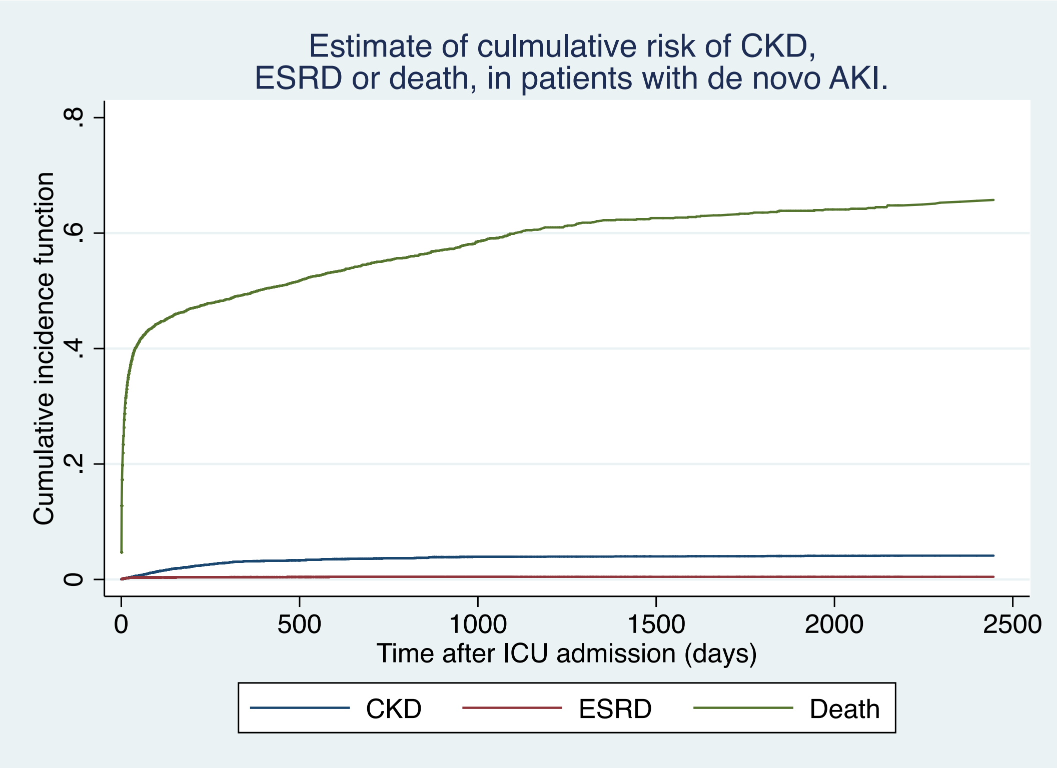

Figure 1. Estimates of cumulative risk of developing CKD, ESRD or of death in patients with de novo AKI.

CKD= Chronic Kidney disease· ESRD= End Stage Renal disease· AKI= Acute kidney Injury·
